# Effective linkage from point of HIV testing to care and treatment in Tanga region, Tanzania

**DOI:** 10.1371/journal.pone.0201644

**Published:** 2018-08-16

**Authors:** David Elias Kayabu, James Samwel Ngocho, Blandina Theophil Mmbaga

**Affiliations:** 1 Clinton Health Access Initiative, Dar es Salaam, Tanzania; 2 Kilimanjaro Christian Medical Centre Duke University Collaboration, Moshi, Tanzania; 3 Kilimanjaro Christian Clinical Research Institute, Moshi, Tanzania; Brighton and Sussex Medical School, UNITED KINGDOM

## Abstract

**Background:**

Linkage to care and treatment is an important part of efforts to accelerate HIV prevention, treatment, care, and support. It offers an opportunity for PLHIV to receive information and services in a timely manner. Clients who present late for HIV care and treatment may miss out on timely initiation of prophylaxis and ART, which may accelerate disease progression and lead to an increased rate of HIV transmission within the community. The objective of this study was to determine the factors influencing effective linkages of newly diagnosed PLHIV from the point of testing to entry in care and treatment centres (CTCs) in Tanga Region, Tanzania.

**Methods:**

This cross-sectional study examined five clinics with a high volume of clients in each of the three districts in Tanga Region. All adults ages 18 years and above at the time of CTC enrolment, between 2010 and 2014, were eligible to participate in the study.

The study engaged both secondary and primary data. To complement the secondary data, mixed methods were applied in primary data collection. Using a structured questionnaire, interviews with the sampled CTC clients while focus group discussions with healthcare providers and in-depth interviews with CTC clients.

The qualitative data were analysed using a thematic analysis framework. The outcome of interest was whether a client enrolled in a CTC within three months of his or her first positive HIV test. A logistic regression model was used to determine factors associated with effective linkage of newly diagnosed HIV clients to CTC.

**Results:**

A total of 16,041 adults from the three study districts were enrolled at a CTC from 2010 to 2014. A total of 1,096 clients from the sampled CTCs were recruited into the study for interview. The characteristics of these clients were representative of the larger group (16,041). The majority (72.4%) were female. More than half (52.1%) were married, and almost a quarter (21.2%) were single. The majority (59.6%) of participants completed primary education and almost half (45.1%) were subsistence farmers. The median CD4 count at enrolment was 218 (87–397) cells/mL with more than half (56.3%) having CD4 counts of less than 350 cells per millilitre (mL). Nearly all (91%) of the clients presented at a CTC within three months of receiving a positive HIV test. In a multivariate analysis, factors that remained significantly associated with early entry in CTC were level of education, CD4 count, and point of diagnosis. Participants’ responses were consistent with many of the factors explained by participants to be barriers to effective linkages and referrals repeated in the FGDs and IDIs across the study sites. For instance, FGD respondent expressed that clients were worried about stigma from their relatives, which creates a delay in seeking treatment.

**Conclusion:**

Although the rate of early entry in care and treatment services is high, surprisingly was a marked increase in those who waited more than three months to seek treatment. To meet the target, issues such as disclosure and stigma need to be addressed.

## Introduction

HIV testing and counselling services in developing countries have expanded rapidly to ensure that PLHIV receive ART and that ART is initiated at the right time [1]. The rapidly expanding HIV testing services can only be realized if individuals diagnosed with HIV are subsequently linked to and enrolled in HIV care and treatment programs in a timely manner. In 2012, 62 percent of women and 47 percent of men ages 15–49 had ever been tested for HIV and received their test results, and only 30 percent of women and 27 percent of men had been tested and received the results in the 12 months before the survey [2].

By the end of 2011, it was estimated that only 65 percent of adults and 35 percent of children who needed ART in Tanzania were receiving it, which indicates that further efforts are needed to reach all those in need of HIV treatment [3]. In many settings, HIV testing services are much more numerous than CTCs. Clients diagnosed by HIV testing points are expected to be referred and enrolled in a CTC. As part of the move to accelerate HIV prevention, treatment, care, and support, strong linkages to care and treatment are needed for PLHIV to receive available services in a timely manner. Poor linkage has been identified as a contributory factor to late enrolment into HIV care and treatment. Clients who present late for HIV care and treatment miss the opportunity for timely initiation of prophylaxis against opportunistic infections; they may experience more rapid disease progression, are more likely to transmit HIV to a sexual partner, and face a greater likelihood of dying [4,5].

Despite the integration of HTC with other health services, linkages between the various points of diagnosis (voluntary testing and counselling, provider-initiated testing and counselling, prevention of mother-to-child transmission of HIV [PMTCT], early infant diagnosis [EID], tuberculosis [TB] clinics, etc.) and enrolment into care, treatment, and support services are poor. This challenge has been reported in both developed and developing countries, where numerous newly diagnosed HIV-positive clients present late for enrolment into CTCs [6,7].

Developing evidence from studies involved in referring people diagnosed with HIV to HIV clinics in Tanzania shows that as few as 14 percent of referrals are successful if the linkage system is ineffective [8]. However, in settings where there are effective linkage systems across the HIV care continuum, the rate of completed referrals between the various points of diagnosis and HIV treatment clinics is high. For example, a study conducted in rural northwestern Tanzania reported uptake rates for care and treatment of 72 percent and 66 percent (p-value = 0.27) among men and women, respectively, within six months of their HIV testing and referral to care and treatment.

About a quarter (23%) [9] of PLHIV present late to care and treatment. To a large extent, this late presentation is associated with a poor linkage mechanism. The factors associated with effective linkages are poorly understood in Tanzania. Therefore, this research study aimed to explore the linkage mechanism and determining factors associated with effective linkages and uptake to care and treatment after HTC.

To maximize the benefits of HTC, more efforts are needed to ensure that all clients diagnosed with HIV are linked to CTCs in a timely manner. Systemic factors, such as the following, are the reasons reported, most often, for poorly functioning linkage systems: inadequate training of healthcare providers, poor quality linkage or referral letters, and a lack of feedback between facilities [10,11]. Client-level factors affecting uptake to care and treatment include economic costs and preferences for, or closeness to, certain facilities [10]. Although community factors probably affect linkages, too, published data on this and other factors is limited. Therefore, in this study, we investigated factors influencing effective linkages to care and treatment services for PLHIV, both at the facility level and the client level. A mixed-methods approach allowed us to develop an in-depth understanding of the factors we identified. Findings from this study will inform future efforts to improve the client linkage and referral process to access HIV care and treatment.

## Methods

### Study design

We conducted a cross-sectional study involving clients at health facilities in the three study districts. All adults, ages 18 years and above at the time of data collection, were invited to participate in the study. The study enrolled clients from the fifteen study clinics who were registered in care and treatment from 2010 to 2014. We used both qualitative and quantitative methods for data collection. More importantly, qualitative added in-depth understanding of the factors identified in quantitative.

### Data sources

The study used both primary and secondary data. For primary data, client information was collected using an interview schedule (quantitative) and an interview guide (qualitative). For the quantitative data collection, interview questions focused on four areas: demographic data, socioeconomic data, psychosocial perceptions of HIV care and treatment services, and knowledge related to HIV care and treatment services. Face-to-face interviews were conducted by trained research assistants to collect data from study participants. To preserve confidentiality, study participants were identified using their unique CTC ID number.

We conducted a total of three FGDs in the two districts; two in Tanga CC (at Bombo Hospital and Ngamiani Health Center), and one in Korogwe DC (at Handeni Hospital). With five to eight health care providers from different units within the facility. Providers were those working in CTC, TB unit, and reproductive and child health (RCH) clinics. In addition, we conducted 25 in-depth interviews (IDIs) with people living with HIV attending clinic in the selected facilities. Eleven in Tanga Urban, 7 in Handeni, and 7 in Korogwe).

Although they used an interview guide to direct the conversation, when needed, interviewers probed further, to gain a more in-depth understanding of the factors influencing effective linkages to care and treatment. All interviews were in the Kiswahili language. Interviewers encouraged participants to narrate their experiences and asked clients to give their opinion on ways to improve linkages. Interviews were audio-recorded with participants’ permission.

Secondary data were gathered from routine data collection in CTCs. This client-level data was collected using standard national client monitoring system tools known as CTC2. CTC2 is a client record form that stays at the clinic. It contains the client’s unique CTC ID number and records all visits that a client makes to the clinic. The CTC2 card is the main source of data entered in the CTC2 database, the national HIV and AIDS care and treatment database where client-level data is stored and used to produce reports. The database has been designed according to the National AIDS Control Programme’s (NACP) standard CTC2 form to capture all collected information. The two main data tables, which include the client table and visits table, were used in the analysis. The information contained in the client table is documented immediately at enrolment time, and the information in the visits table is collected regularly when a client attends the clinic.

#### Variables in the client table

Regional district codes, division name, village name, client unique ID, date of birth, date first positive HIV test, date confirmed HIV-positive, referred from, marital status, drug allergies, community support group, prior exposure, transfer in ID, tuberculosis (TB) ID, home-based care, client code, height.

#### Variables in the visits table

Client’s unique ID, visit date, weight, now pregnant, TB screening, antiretroviral (ARV) status code, ARV reason code, ARV code, CD4 count, CD4%, nutritional status, relevant co-medication, haemoglobin (Hb), height, WHO stage, functional status, ARV adherent, eligibility reason, visit type code.

### Sample size and sampling method

#### Sample size estimation

Sample size was calculated on a wealth index constructed from the economic data gathered, but the calculations could be applied to other factors in the questionnaire. We assumed that among the clients that come to a CTC within three months of receiving their first positive HIV test result, 40 percent are considered wealthy and 30 percent are not. To detect the difference of 10 percent with 80 percent power and 5 percent significance, a simple random sample of 750 clients was needed. With the expected clustering in clinics, we assumed a design effect of two. Therefore, a sample size of 1500 clients was calculated to achieve 80 percent power at 5 percent significance to detect a 10 percent difference in the proportion of those who attend CTC within three months of their first HIV diagnosis.

We had no predetermined sample size for a qualitative part of this study, participants were recruited until when the saturation point was reached.

#### Sampling method

A list of health facilities considered to have a high volume of clients was first identified in each district. Then a simple random sampling selected five CTCs per district. Priority of selection was given to the CTCs considered to have a high volume of clients and then to the health facilities close to having a high volume of clients. Following that, a total of 15 CTCs from three districts (five per district) were identified. From each selected CTC, at least 50 attendees who met the study inclusion criteria during the survey period (two months) were invited to participate. Then everyday five clients were selected randomly from the daily client’s appointment logbook. The same process was repeated every day until the planned 50 clients per clinic were enrolled. While for qualitative part, a convenience method was used for recruiting study participants for the FGDs and IDIs.

#### Data management and analysis

Qualitative data were analysed using a thematic analysis framework with six main steps: familiarizing oneself with the data, generating initial codes, searching for themes, reviewing themes, defining and naming themes, and producing the report (Braun & Clarke, 2006). To capitalize on validity and reliability, the collected information was given a code for confidentiality. Field notes were expanded into full sentences and paragraphs by adding details of what was observed using the notetaker’s notes and audio recordings. The expanded notes were translated into English, typed in Microsoft Word 2007, and stored on a computer. Narratives were read, coded, and cross-checked. Six main themes emerged from the data: referral letter/forms, escorting clients, stigma, falling sick, denial of results, and counselling procedures.

Data collected through quantitative methods were entered in an Access database (interfaced by Epi Info3.5.4). The database was developed to correspond to the study questionnaire. The data were cleaned for quality control purposes and transferred into Stata format to form a primary data set. The data from the CTC2 databases were extracted based on the study variables and transferred into Stata format to form a secondary data set. The two data sets (the one obtained from primary data collection and the other from the CTC2 database) were then merged using a unique CTC ID number to form a single data set.

The outcome of interest in this study was whether a client enrolled at a CTC within three months of the date of his or her first HIV-positive test. The period was calculated by subtracting the date of registration at a CTC from the date of the client’s first positive HIV test. Descriptive analyses were used to capture different characteristics of the study participants. Continuous variables were described in terms of median and interquartile range, and categorical variables were described in terms of frequencies and percentages.

World Health Organization (WHO) clinical stages and CD4 counts were used to document the disease progression between HIV clients presenting late at CTCs and those presenting early. The changes in baseline and follow-up were determined for both the WHO clinical stages and CD4 counts, and these were compared between the groups. In documenting the disease progression, the proportion of HIV clients in advanced clinical stages (i.e., WHO clinical stages III and IV) was determined in both groups (i.e., for those presenting late at CTCs and those presenting early) at baseline, six months, and 12 months. Two kinds of analyses were carried out to describe the disease progression, in terms of CD4 counts, for the two groups. The first analysis examined the disease progression based on the proportion of HIV clients who have CD4 counts of less than 350 at baseline, six months, and 12 months and compared the two groups, and the second analysis compared the disease progression in the two groups in terms of median CD4 counts at baseline, six months, and 12 months. The analysis was carried out to HIV clients in the study districts who had both WHO clinical stages and CD4 counts at baseline, 6month and 12months.

Cross tabulations and crude odds ratios were conducted to assess preliminary associations between independent variables and the outcome of interest. Bivariate analysis assessed potential confounders between variables that were statistically significant in the univariate analyses. The relevant confounders were ascertained when the estimate/odds ratio changed by 20 percent or more. Following the assessment, no relevant confounders were identified. Multivariable logistic regressions were conducted for the factors that were statistically significant from the univariate analyses. The final model was chosen by performing the stepwise model selection by Akaike Information Criterion (AIC). And for time changing variables including age, marital status, and level of education, the baseline data was used. The clustering effect was taken into consideration to adjust for variations within and between clinics. All the analyses were carried out using STATA Version 12 with the results summarized and presented in tables and figures.

### Ethical considerations

The study was approved by the Kilimanjaro Christian Medical University College Research Ethics Committee. Participants signed informed consent forms prior to their involvement in the study. Permission to use Tanga Region’s CTC data were sought at NACP. To maintain confidentiality, unique identifiers were used in lieu of participant names.

## Results

### Characteristics of HIV clients enrolled at CTC in three study districts

In the three study districts, 16,041 adult HIV clients were enrolled at CTCs from 2010 to 2014. Most (72.6%) of the participants were female. The median age was 37. The age group 18–24 years had the fewest clients (8.7%). Half (50.9%) of the participants were married, and about a quarter (26.4%) were single. Most (56.2%) of the clients were from Tanga CC, followed by Korogwe (30.1%). At enrolment, 11.5 percent of participants had CD4 counts of less than 50 cells/mL and 10.8 percent had CD4 counts of 500 cells/mL or more. The WHO stages at enrolment were almost equally represented except for stage 4 (10.8%). See Table 1 below for details.

**Table 1:** Characteristics of HIV clients enrolled at CTCs in the three study districts (N = 16,041) and sampled study participants (n = 1,096).

Variable	Characteristics	N (%)	n (%)
			
**Sex**			
	Male	4,403 (27.4)	302 (27.6)
	Female	11,638 (72.6)	794 (72.4)
**Age**			
	18–24	1,391 (8.7)	58 (5.3)
	25–34	5,369 (33.5)	269 (24.5)
	35–44	5,233 (32.6)	420 (38.3)
	45+	4,048 (25.2)	349 (31.8)
**Marital status**			
	Single	4,228 (26.4)	232 (21.2)
	Married	8,171 (50.9)	571 (52.1)
	Cohabiting	320 (2.0)	21 (1.9)
	Divorced	1,991 (12.4)	148 (13.5)
	Widowed	1,171 (7.3)	112 (10.2)
	Data unavailable	160 (1.0)	12 (1.1)
**Referred from**			
	RCH/PMTCT/EID	1,232 (7.7)	52 (4.7)
	In-client department	1,170 (7.3)	155 (14.1)
	Out-client department	971 (6.1)	111 (10.1)
	Home-based care	47 (0.3)	3 (0.3)
	PLHIV group	10 (0.1)	0 (0.0)
	Sexually transmitted infection	37 (0.2)	4 (0.4)
	TB	316 (2.0)	17 (1.5)
	VCT	10,746 (67.0)	666 (60.8)
	Other	466 (2.9)	24 (2.2)
	Missing	1,047 (6.5)	64 (5.8)
**District**			
	Tanga CC	9,023 (56.2)	466 (42.5)
	Korogwe DC	4,822 (30.1)	386 (35.2)
	Handeni DC	2,197 (13.7)	244 (22.3)
**CD4 count**			
	<50	1851 (11.5)	116 (10.6)
	50–199	3251 (20.3)	280 (25.6)
	200–349	2377 (14.8)	219 (20.0)
	350–499	1553 (9.7)	120 (10.9)
	500+	1733 (10.8)	86 (7.8)
	Missing	5276 (32.9)	275 (25.1)
**WHO stage**			
	I	5022 (31.3)	354 (32.3)
	II	4228 (26.4)	322 (29.4)
	III	4929 (30.7)	310 (28.3)
	IV	1729 (10.8)	102 (9.3)
	Missing	133 (0.8)	8 (0.7)
**Year of enrolment**			
	2010	3,418 (21.3)	188 (17.2)
	2011	3,257 (20.3)	211 (19.2)
	2012	2,878 (17.9)	210 (19.2)
	2013	3,077 (19.2)	223 (20.3)
	2014	3,412 (21.3)	264 (24.1)

### Time elapsed from the first positive HIV test to entry in HIV care

**Fig 1:**
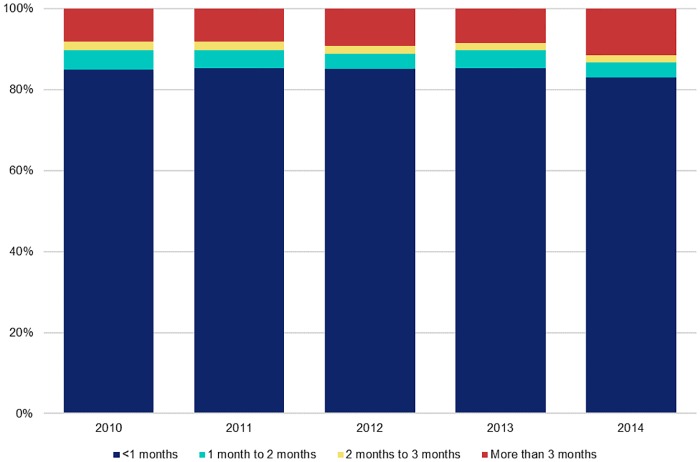
The trend of CTC enrolment by the duration of entry from 2010 to 2014 (N = 16,041).

Of the 16,041 HIV clients enrolled at CTCs in the study districts, about 91 percent entered within the first three months of their HIV-positive diagnosis, with the largest proportion enrolling in the first month, across all years of the study period. This trend remained consistent from 2010 to 2014. However, in 2014 there was a slight drop in the proportion of HIV clients who enrolled at a CTC within the first month of receiving their HIV-positive diagnosis (82.9% in 2014 versus 85.3% in 2013). Moreover, the percentage of HIV clients enrolling at a CTC more than three months after receiving their HIV-positive diagnosis increased over time, from 8.2% in 2010 to 11.6% in 2014 (Fig 1).

### Characteristics of study participants from quantitative interviews

A total of 1,096 HIV clients from the sampled CTCs were recruited into the study for interviews. The characteristics of these clients were representative of the larger group. The majority (72.4%) were female. The median age at enrolment in CTC was 40 years. The age group 18–24 years had the fewest (5.3%) clients. More than half (52.1%) of participants were married, and almost a quarter (21.2%) were single. The majority (59.6%) of participants had completed primary education, more than two-thirds (69.1%) were Muslim, and almost half (45.1%) were involved in subsistence farming. The majority (45.1%) of the participants didn’t know their monthly income status; but 15.2 percent had monthly incomes of less than 40,000TZS, and 15 percent had monthly incomes between 80,000TZS and 150,000TZS. The travel time to a CTC was less than 30 minutes for almost half (48.1%) of the study participants. At CTC enrolment, more than half of participants (56.3%) had CD4 counts less than 350 cells/mL, and few (9.3%) were in WHO clinical stage IV. The majority (24.1%) of the study participants had enrolled in 2014, with almost equal distribution in the other four years. Most were from Tanga CC (42.5%) followed by Korogwe DC (35.2%) and Handeni DC (22.3%). A referral letter was the major mechanism for referring clients to a CTC (81.0%), with the majority having been referred from VCT (60.8%). Only 4.7 percent were referred from RCH/PMTCT. Most (59.7%) were the breadwinners in their family, and most (61.5%) were the primary decision makers, as well. Nearly all (93.5%) had disclosed their HIV status, with many having disclosed to relatives (30.6%) and partners (24.8%). Among married participants, nearly half (40.6%) were counselled and tested for HIV’ together. See Table 2 below for details.

**Table 2:** Characteristics of study participants by time of entry in CTC (n = 1,096).

Variable	Total (%)	Entry <1 month	Entry <2 months	Entry <3 months
		Yes (%)	Yes (%)	Yes (%)
Total	1,096	943 (86.0)	992 (90.5)	1019 (93.0)
**Sex**				
Male	302 (27.6)	271 (89.7)	281 (93.1)	287 (95.0)
Female	794 (72.4)	672 (84.6)	711 (89.6)	732 (92.2)
**Age**				
18–24	58 (5.3)	45 (77.6)	52 (89.7)	54 (93.1)
25–34	269 (24.5)	220 (81.8)	235 (87.4)	247 (91.8)
35–44	420 (38.3)	362 (86.2)	378 (90.0)	387 (92.1)
45+	349 (31.8)	316 (90.5)	327 (93.7)	331 (94,8)
**Marital status**				
Single	232 (21.2)	197 (84.9)	208 (89.7)	214 (92.2)
Married	571 (52.1)	482 (84.4)	514 (90.0)	531 (93.0)
Cohabiting	21 (1.9)	20 (95.2)	20 (95.2)	20 (95.2)
Divorced	148 (13.5)	134 (90.5)	136 (91.9)	137 (92.6)
Widowed	112 (10.2)	101 (90.2)	104 (92.9)	106 (94.6)
Missing	12 (1.1)	9 (75.0)	10 (83.3)	11 (91.7)
**Education level**				
None	153 (14.0)	140 (91.5)	148 (96.7)	150 (98.0)
Some primary education	180 (16.4)	155 (86.1)	161 (89.4)	165 (91.7)
Completed primary education	653 (59.6)	560 (85.8)	589 (90.2)	605 (92.7)
Some secondary education	35 (3.2)	29 (82.9)	32 (91.4)	33 (94.3)
Completed secondary education	71 (6.5)	57 (80.3)	60 (84.5)	63 (88.7)
Higher education	0 (0.0)	0 (0.0)	0 (0.0)	0 (0.0)
Missing	4 (0.4)	2 (50.0)	2 (50.0)	3 (75.0)
**Religion**				
Muslim	757 (69.1)	651 (86.0)	686 (90.6)	704 (93.0)
Christian	334 (30.5)	288 (86.2)	302 (90.4)	311 (93.1)
Don’t have religion	1 (0.1)	1 (100.0)	1 (100.0)	1 (100.0)
Missing	4 (0.4)	3 (75.0)	3 (75.0)	3 (75.0)
**Daily activities**				
Student	4 (0.4)	3 (75.0)	4 (100.0)	4 (100.0)
Government or private employee	71 (6.5)	61 (85.9)	64 (90.1)	66 (93.0)
Small scale business	396 (36.1)	339 (85.6)	355 (89.6)	363 (91.7)
Commercial farmer	9 (0.8)	8 (88.9)	8 (88.9)	8 (88.9)
Subsistence farmer	494 (45.1)	429 (86.8)	451 (91.3)	464 (93.9)
None	72 (6.6)	61 (84.7)	67 (93.1)	68 (94.4)
Other	35 (3.2)	30 (85.7)	31 (88.6)	32 (91.4)
Missing	15 (1.4)	12 (80.0)	12 (80.0)	14 (93.3)
**Income quintile (per month)**				
Lowest (≤40,000)	167 (15.2)	148 (88.6)	158 (94.6)	158 (94.6)
Second (40,001–80,000)	134 (12.2)	118 (88.1)	124 (92.5)	126 (94.0)
Middle (80,001–150,000)	164 (15.0)	134 (81.7)	142 (86.6)	146 (89.0)
Highest (≥150,001)	128 (11.7)	98 (76.6)	106 (82.8)	113 (88.3)
Don’t know	472 (43.1)	417 (88.3)	433 (91.7)	446 (94.5)
Missing	31 (2.8)	28 (90.3)	29 (93.5)	30 (96.8)
**Family breadwinner**				
Study participant	654 (59.7)	577 (88.2)	601 (91.9)	611 (93.4)
Others	436 (39.8)	361 (82.8)	386 (88.5)	402 (92.2)
Missing	6 (0.6)	5 (83.3)	5 (83.3)	6 (100.0)
**Decision maker in the family**				
Study participant	674 (61.5)	594 (88.1)	618 (91.7)	631 (93.6)
Others	418 (38.1)	346 (82.8)	371 (88.8)	384 (91.9)
Missing	4 (0.4)	3 (75.0)	3 (75.0)	4 (100.0)
**Transport type to CTC**				
Walking	321 (29.3)	272 (84.7)	287 (89.4)	296 (92.2)
Bicycle	77 (7.0)	69 (89.6)	71 (92.2)	74 (96.1)
Motorcycle/bajaj	221 (20.2)	188 (85.1)	198 (89.6)	203 (91.9)
Public transport	431 (39.3)	377 (87.5)	396 (91.9)	406 (94.2)
Private car	34 (3.1)	26 (76.5)	29 (85.3)	29 (85.3)
Others	7 (0.6)	6 (85.7)	6 (85.7)	6 (85.7)
Missing	5 (0.5)	5 (100.0)	5 (100.0)	5 (100.0)
**Travel duration to CTC**				
Less than 30 minutes	532 (48.5)	460 (86.5)	481 (90.4)	494 (92.9)
30 minutes to one hour	323 (29.5)	274 (84.8)	289 (89.5)	297 (91.9)
More than one hour	238 (21.7)	206 (86.6)	219 (92.0)	225 (94.5)
Missing	3 (0.3)	3 (100.0)	3 (100.0)	3 (100.0)
**Partner registered for CTC**				
Yes	328 (38.5)	281 (85.7)	299 (91.2)	309 (94.2)
No	334 (39.2)	290 (86.8)	303 (90.7)	308 (92.2)
Don’t know	154 (18.1)	136 (88.3)	140 (90.9)	145 (94.2)
Missing	36 (4.3)	30 (83.3)	32 (88.9)	32 (88.9)
**Live near CTC**				
Yes	798 (72.8)	691 (86.6)	726 (91.0)	743 (93.1)
No	296 (27.0)	250 (84.5)	264 (89.2)	274 (92.6)
Missing	2 (0.2)	2 (100.0)	2 (100.0)	2 (100.0)
**Reason for attending distant CTC**				
Better service	171 (57.8)	142 (83.0)	150 (87.7)	156 (91.2)
Fear of meeting people I know	67 (22.6)	60 (89.6)	63 (94.0)	66 (98.5)
Referred here	33 (11.2)	26 (78.8)	29 (87.9)	30 (90.9)
Others	21 (7.1)	18 (85.7)	18 (85.7)	18 (85.7)
Missing	4 (1.4)	4 (100.0)	4 (100.0)	4 (100.0)
**CD4 count**				
<50	116 (10.6)	104 (89.7)	108 (93.1)	111 (95.7)
50–199	280 (25.6)	253 (90.4)	264 (94.3)	267 (95.4)
200–349	219 (20.0)	187 (85.4)	198 (90.4)	204 (93.2)
350–499	120 (10.9)	105 (87.5)	108 (90.0)	112 (93.3)
500+	86 (7.8)	65 (75.6)	72 (83.7)	74 (86.1)
Missing	275 (25.1)	229 (83.3)	242 (88.0)	251 (91.3)
**WHO stage**				
I	354 (32.3)	297 (83.9)	315 (89.0)	326 (92.1)
II	322 (29.4)	281 (87.3)	295 (91.6)	301 (93.5)
III	310 (28.3)	275 (88.7)	289 (93.2)	294 (94.8)
IV	102 (9.3)	85 (83.3)	88 (86.3)	93 (91.2)
Missing	8 (0.7)	5 (62.5)	5 (62.5)	5 (62.5)

### Factors associated with early entry in CTC of newly diagnosed HIV clients

In a multivariate analysis, factors that remained significantly associated with early entry in CTC were level of education, CD4 count, and point of diagnosis. Those who had no formal education were more likely to have early entry in a CTC than were those who completed secondary education. Participants with CD4 counts of <50 and 50–199 cells/mL had higher odds of early entry in a CTC than did those who had a CD4 count of >500 cells/mL. Participants enrolled in CTCs in 2013 were more likely to have early entry than were those enrolled in 2010. See Table 3 for details.

**Table 3:** Univariate and multivariate analysis of factors associated with early entry in CTC.

Variable	Univariable			Multivariable		
	Odds ratio	95% CI	P-value	Odds ratio	95% CI	P-value
**Sex**						
Male	1.00			1.00	(reference)	
Female	0.63	0.41–0.96	0.031	0.52	0.22–1.22	0.132
**Age**						
18–24	1.00	(reference)		1.00	(reference)	
25–34	1.30	0.65–2.59	0.460	0.30	0.05–1.64	0.164
35–44	1.80	0.92–3.55	0.088	0.45	0.08–2.39	0.348
45+	2.77	1.35–5.65	0.005	0.50	0.09–2.86	0.439
**Marital status**						
Single	1.00	(reference)				
Married	0.96	0.63–1.47	0.859			
Cohabiting	3.55	0.46–27.33	0.223			
Divorced	1.70	0.88–3.28	0.114			
Widowed	1.63	0.80–3.35	0.182			
**Education level**						
None	2.65	1.17–5.98	0.019	14.16	1.45–138.12	0.023
Not completed pr. Education	1.52	0.74–3.13	0.253	1.58	0.50–5.05	0.439
Completed pr. Education	1.48	0.79–2.76	0.219	1.69	0.62–4.65	0.307
Not completed sec. education	1.19	0.41–3.41	0.750	2.06	0.21–19.85	0.533
Completed sec. education	1.00	(reference)		1.00	(reference)	
**CD4 count**						
<50	2.80	1.29–6.07	0.009	3.64	0.98–13.50	0.053
50–199	3.03	1.61–5.70	0.001	3.11	1.14–8.50	0.027
200–349	1.89	1.02–3.50	0.044	1.78	0.68–4.66	0.241
350–499	2.26	1.09–4.70	0.029	1.76	0.59–5.24	0.314
500+	1.00	(reference)		1.00	(reference)	
**WHO stage**						
I	1.00	(reference)				
II	1.32	0.85–2.03	0.215			
III	1.51	0.96–2.37	0.075			
IV	0.96	0.53–1.74	0.892			
**Year of enrolment**						
2010	1.00	(reference)		1.00	(reference)	
2011	1.19	0.68–2.09	0.539	2.61	0.82–8.31	0.105
2012	0.98	0.57–1.68	0.937	0.69	0.–1.76	0.435
2013	1.22	0.70–2.13	0.483	6.97	1.33–36.59	0.022
2014	1.23	0.72–2.11	0.442	0.79	0.29–2.16	0.647
**Referred from**						
RCH/PMTCT/EID	0.35	0.18–0.67	0.002	0.78	0.20–2.97	0.716
In-client department	1.05	0.60–1.83	0.875	0.83	0.32–2.22	0.723
Out-client department	0.82	0.46–1.49	0.524	0.97	0.31–3.06	0.963
Sexually transmitted infection	0.39	0.04–3.76	0.413	0.08	0.003–2.00	0.123
TB	2.06	0.27–15.76	0.486	^a^	^a^	^a^
VCT	1.00	(reference)		1.00	(reference)	
Other	0.11	0.05–0.25	<0.001	0.05	0.02–0.18	<0.001

^a^ *Dropped due to few observations*

### Disease progression

A total of 595 clients had complete data on WHO clinical stage and CD4 count up to 12 months of follow up and were evaluated for HIV disease progression. There was a statistically significant difference in CTC enrolment between HIV clients who presented late and those who presented early, and more than half of the clients who presented late were in stage III or IV (50.8%; 95% CI [46.8%–54.8%]). A smaller proportion of those who presented early were stage III or IV (42.7%; 95% CI [41.3%–44.0%]). However, in the follow-up periods, there was no statistical difference between the two groups at six months and 12 months, respectively.

**Fig 2:**
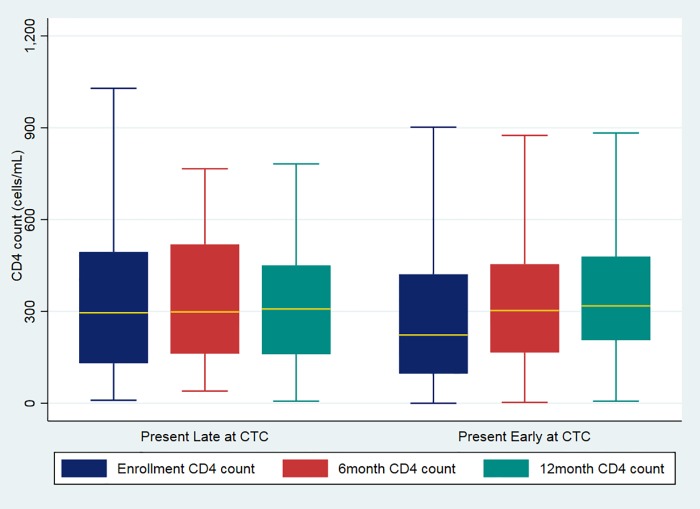
Enrolment and follow-up CD4 counts between HIV clients who presented late and those who presented early at CTC.

The disease progression in relation to CD4 counts showed that at CTC enrolment, the median CD4 count was higher among HIV clients who presented late at CTC [295.5 (131–494)] than among those who presented early (223 [97–421]). However, at both six- and 12-month follow-ups, there was a slight increase in median CD4 counts among clients who presented early at a CTC in comparison with those who presented late (Fig 2).

### Qualitative results

Three FGDs and 25 IDIs were conducted with a total of 45 participants. In the study, saturation was reached in all districts. The male and female participants ranged in age from 20–68 years, with the majority falling in the 40–49 age range. Most participants (53%) were from Tanga CC and had at least a primary-level education, with many holding a tertiary-level education (post-secondary school). One-third of the participants were business men or women; nearly half were healthcare workers; a fifth were farmers; and only one participant was a college student.

#### Factors associated with effective linkages of newly diagnosed HIV clients to CTCs

All participants in the study were aware of HIV-related information on linkage strategies and referral systems. Participants’ responses were consistent with many of the factors explained by participants to be barriers to effective linkages and referrals repeated in the FGDs and IDIs across the study sites.

#### Referrals

Referral forms, or referral letters as some called them, were noted by all FGD participants as being the main method used to link new clients to CTCs. According to their responses, this form/letter was designed by the Tanzanian Ministry of Health Community Gender Elder and Children (MOHCGEC) and sent to all health facilities that have a CTC. After a client is found to be HIV-positive, the form is filled in and addressed to the referral health facility chosen by the client. A respondent from Bombo.

Regional Hospital said:

*The method used is still the same. After a patient tests positive and he or she decides which VCT centre to get treatment, let’s say he or she says, ‘I want to be treated at Bombo CTC,’ so you must give him the letter*.

Another respondent said, before this form is filled out, a client must be asked where he or she comes from so that the client can be easily linked to the facility nearest his or her area of residence.

*First of all, you ask the client that has come to you; you ask him or her where he or she lives, because nowadays HIV medications are available in many centres; you need to ask where that patient lives. He or she tells you, I live in a certain area, and then you mention the CTC which are within his locality*.

There was an insistence on knowing where a client comes from, to facilitate the movement of the client, while accessing care. One respondent from Ngamiani Health Centre said,

*A client goes to VCT; he or she gets tested there. That client came from home and went to get tested; he or she is given a form and explained where he or she is supposed to go, okay? Because at VCT, a client may come to Ngamiani but he or she is from a different district, so he or she will be given a form and asked to go back to where he or she came from*.

It was also reported that at times there are linkages based on departments within a health facility that link a client from one point to another, based on the nature of the disease found.

*Now for example at the RCH, they have a special form which they use; when a patient is diagnosed with HIV, there is a form which is filled and given to a patient to take to CTC*.

However, some linkages for newly identified clients are from people other than healthcare workers and do not involve referral forms. One such client reported being linked by a friend. Another participant, from Bombo Regional Hospital, reported,

*It is my mother who linked me with a CTC because she is also HIV-positive, and I was born with HIV*.

She continued,

*To be honest, at first, because I work at a hospital, first I tested myself and saw the results. Immediately you know that situation, I mean I got worried. After getting worried, my colleagues counselled me and I came here at CTC to start medication*.

Many said a problem with the referral forms is that they lack important information that would allow healthcare workers to follow up easily with clients who have tested HIV-positive. They suggested that the MOHCGEC modify the form to include other important information that can be helpful when looking for an HIV-positive client when he or she does not comply with attending CTC.

#### Escorting clients

Escorting clients was one of the ways proposed in the FGDs to link new clients to care and treatment. According to the respondents, this is done based on the client’s level of understanding and is mostly used when a client has chosen to get care within the same healthy facility:

*Every time you explain to him or her to come to CTC and he or she does not understand, maybe he or she asks to be escorted, then you can decide to take him or her; but in most cases, we just give them the referral letters, and they go to the CTC of their choice*.

Escorting new clients came about because clients were not attending the CTC to which they had been referred. One respondent said,

*At first, we saw that there was a problem: that clients did not go to the CTC which they were referred to. So, we used that method (escorting), especially those who were from the reproductive and child health clinic*.

During FGDs respondents expressed that escorting a client is fine if the client requests it, but an escort is required if the client is unconscious. For IDIs, most of the respondents thought an escort was the best referral mechanism, because most of them did not want their status to be easily disclosed to other people—even family members who may not know they are HIV-positive or even that they are on ART. The common suggestion was for health service staff to be available always so clients can choose where to get services.

#### Stigma

Procedurally, whenever a client tests HIV-positive, there must be a referral to the CTC nearest his or her place of residence, to make it easy for him or her to get treatment. However, even with this protocol in place, some clients have not been attending, or have delayed attendance at, a CTC due to various factors such as stigma. Stigma was evident both internally (with self-stigma, by the client) and externally (with stigma from relatives, healthcare workers, and the community). An FGD respondent from Handeni Hospital said:

*Most who come to get tested are convinced by their friends or relatives. They say, ‘let’s go get tested; let’s go get tested.’ When he or she is tested, and found to be HIV-positive, he or she is with them, this person is not ready to let the colleagues that came with him or her to know his or her status at that time, so even if you give him or her the letter, he or she will not come*.

Another FGD respondent expressed that clients may be worried about stigma from their relatives, which creates a delay in seeking treatment:

*One may think ‘if I disclose my status, how will my relatives think of me? How will I start telling them that I did go the hospital*?

According to a respondent, stigma from the community also presents a problem:

*Another factor could be fear of how the society will treat it based on the reality that he or she is infected with HIV, when one thinks about the stigma that may come from the relatives or family, he or she may decide not to disclose his or her status*.

Because of stigma, most clients prefer to travel a longer distance to obtain their care and treatment than visit their nearest facility to avoid the risk of encountering people who might recognize them and find out their HIV status.

#### Falling sick

Falling sick was brought up by a respondent as a factor that delays new clients from attending a CTC after they are referred:

*It is the short-sighted mind that I mentioned. A person may decide, to wait and see what will happen. Will I get sick or not? If they don’t get sick, they believe they are not infected but if they get sick, then they believe that they are really infected with HIV*.

The habit of waiting to fall sick before seeking treatment is often a fatal decision, because the person’s health has already deteriorated by the time the individual reaches the hospital. One respondent explained,

*[They give themselves] hope that, maybe it is not true; let me wait and see how it goes. Because they delay too much in seeking care, that is why by the time they come, they are really worn out (really sick). They say, let me wait and see the end, what is the end? They come to the hospital too sick that some cannot even walk. They have to be assisted by their relatives*.

The decision to wait until the person falls sick is related to other reasons that respondents gave for putting off seeking treatment, such as denial of results and feeling well even after testing HIV-positive. By the time these clients accept their health condition, it is often too late for treatment. The discussion also revealed that most of the clients do not understand the consequences of starting treatment late. If they knew the severity of that decision, presumably, fewer would wait to get treatment.

#### Denial of results

According to respondents, most clients delay following through on their referral to a CTC, because they are in denial of their HIV-positive test results. Emotionally, they may not be able to face an HIV diagnosis, or they may believe that the results given to them were false. One informant said,

*Some patients come to test for HIV out of planning, someone sits down and decides to go and test and if they find they are positive they run away and reject the results given to them. At the end of the day they delay attending to CTC for more treatment*.

Likewise, an IDI respondent explained,

*Some maybe don’t believe they are infected. They do not believe that they actually have HIV, and some may think, if I have HIV, why should I go to the hospital before infecting other people*.

Another said,

*The client may not have expected to be diagnosed with HIV, and most who come for tests, do not expect to be diagnosed HIV-positive. So, when some test and are diagnosed HIV-positive, some understand and accept it, but for some, it is not that easy. That is why some would need to go back home and think about it (their newly diagnosed HIV-positive status), and they tell you for now, even if you have given me a referral letter, let me go home first and think about it, prepare myself, and see if I am ready to start CTC or not. So, for many it is how they receive the results which makes them not come directly to CTC but instead go home first*.

#### Counselling Techniques

Knowledge and competency of the counselling officer could also contribute to delays, as some may not clearly convey the consequences of failing to attend a CTC after the client has tested positive for HIV.

*It depends on the counsellor that he or she will meet with. If he or she meets a counsellor who is not competent, he or she may not focus a lot on telling the patient to go to CTC, because he or she looks healthy; he or she does not have any symptoms of HIV infection. He or she may thus wait until he or she gets symptoms before going to CTC*.

Most of the PLHIV said that some healthcare providers do not provide detailed counselling. Instead, they give the results and ask for the nearest health facility, to address the referral form. This is done as a formality and is sometimes a result of having too many clients to attend to. Another respondent said that they are given group counselling, without consideration for individual needs and preferences.

## Discussion

We found that most participants entered care and treatment within one month of their positive HIV diagnosis. However, over the five-year study period, there was a slight drop in the proportion of clients who entered care and treatment within the first month. Entry in care and treatment increases with time, and at three months, the entry in CTC was 91 percent. The reported proportion of entry in care is high compared to other studies. For instance, in northern Tanzania, about 17 percent of HIV-positive clients entered late (>six months) into care and treatment and 6.3 percent were lost to follow-up [12]. A similar low rate of entry into care was found in Kenya, where, among the clients who present to a CTC, 70 percent presented within three months of their diagnosis [13]. The difference might be because of the large sample size in the present study compared to previous studies [14]. In the United States, about 75 percent of adults enter care after a period of six months [14,15]. At four months, enrolment in an HIV CTC among adults in the United States is 72 percent [16]. However, the trend of late entry in care and treatment in our study populations showed a marked increase over time, from 8.2 percent in 2010 to 11.6 percent in 2014. Generally, results show some of the achievements in the national HIV/AIDS control program. Those who test positive are counselled on the importance of enrolling early in treatment. According to these results, if accompanied by a high uptake of ART then Tanzania might achieve the second 90 of the global target of 90-90-90 by 2020: the second 90 aim to provide treatment to 90% of those testing positive [1].

In the present study, the multivariate analysis revealed several significant predictors of early entry in an HIV CTC. Level of education was found to be significantly associated with early entry in care and treatment. Those with no formal education were 14.6 times more likely to enter a CTC early than were those who completed secondary school education. Several studies have reported a similar association between level of education and early entry in care and treatment. For instance, in Kenya, among adult clients enrolled in a CTC, those without a secondary education were 27 percent more likely to enter a clinic than were those who completed secondary education [13]. However, in the United States, a higher educational level was associated with higher odds of early entry in a CTC. There, those with a high-school education and above were 2.8 times more likely to enter care early than were those who had a secondary education or less [17].

The baseline CD4 count level was significantly associated with early entry in care and treatment. Clients with low baseline CD4 counts had higher odds of early entry in care and treatment than did those with high baseline CD4 counts. Those with a CD4 of less than 50 cells/mL were 3.6 times more likely to enter care early than were those who had a CD4 count higher than 500 cells/mL. Similarly, in the United States, the level of a client’s baseline CD4 count was significantly associated with early entry into care and treatment among newly diagnosed HIV-positive adults [18]. In the Netherlands, early entry into care and treatment was not associated with baseline CD4 count [19]. In this study, early entry was defined as enrolment within four weeks after diagnosis. Clients with low CD4 counts might present with some AIDS symptoms and therefore need medical care, whereas those with high CD4 counts consider themselves well and do not feel the need to enrol in care. The problem of many clients failing to seek treatment until they get sick emerged as one of the key themes, in both the FGDs and IDIs.

We found that low median CD4 counts were associated with early entry into care. At the six- and 12-month follow-ups, there was no statistical difference between the median CD4 counts of those who entered early compared to those entered late. This similarity might be because, when clients enter care early, clinical treatment is started early [20] and there is good immunological recovery, which also slows down disease progression. There was a marked increase in median CD4 count among clients who presented early to care. This finding supports the need to enrol tested clients in CTC as early as possible, in line with the 90-90-90 UNAIDS target. Individuals presenting late, and with advanced disease, are less likely to have immune recovery and thus have reduced quality of life and lower life expectancies. Although more than half of clients who presented late had advanced disease (WHO clinical stages III and IV), at the six- and 12-month follow-up periods, there was no statistical difference in WHO clinical staging between the group that presented early and those who presented late. At the time of data collection, the guideline was to initiate antiretroviral therapy with all clients with WHO clinical stage III and IV (HIV/AIDS Treatment Guideline, 2015). Those who presented early but with WHO stage I & II were kept under observation, without ART, until their disease progressed to the advanced stages. This might explain why there is no significant difference at six- and 12-month follow-up. The same was reported in northern Tanzania where more than 50 percent of clients who were ineligible for ART at baseline became eligible after 12 months of follow-up [12].

Point of HIV testing was found to be significantly associated with early entry in care and treatment. Clients who were diagnosed from points other than a health facility and VCT centre had a 5-percent lower chance of early entry in care, compared to those who were diagnosed at a VCT centre. In northern Tanzania, clients who tested in a community setting were less likely to enter care early than were those who tested at a healthcare facility [12]. Similarly, in many studies, clients who were diagnosed at in-patient departments were more likely to enrol in a CTC early than were those diagnosed at out-patient departments or testing facilities [14]. Counselling should stress the importance of early CTC, whether or not the client has a good CD4 count or feels healthy, to allow close monitoring of the client and prophylaxis for opportunistic infections, to avoid a drop in CD4 count and allow timely initiation of medication.

We found that the most common linkage mechanisms were a referral letter, verbal referral, and escorting. There was no association between linkage mechanism and early entry in care. However, in an FGD with healthcare providers, a referral letter was preferred for linkage between different health facilities. For referrals within the same facility, escorting clients was identified as the most effective linkage mechanism. According to one respondent quoted earlier in this report,

*At first, we saw that there was a problem: that clients did not go to the CTC which they were referred to. So, we used that method (escorting), especially those who were from the reproductive and child health clinic*.

Factors such as sex, marital status, income, and distance to the nearest health facility, which in other studies were reported to be significantly associated with early entry in a CTC, had no association in the present study. The reasons might be the small sample used in the previous studies and the definition of early entry in care and treatment. In this study, we defined early entry as up to three months’ postdiagnosis, focusing primarily on the first and third months; whereas, in other studies early entry was defined as the first six months, with a focus on months one, three, four, and six [14,15].

### Strengths and limitations

One key strength of the current study is the use of both qualitative and quantitative data collection methods. The addition of qualitative data gave an in-depth understanding of some of the factors identified in the quantitative analysis.

These results should be interpreted with caution, partly because the total sample included in the analysis is below the minimum required sample size. Evidenced with a wide confidence interval seen in some of the estimates, even though some of the point estimates were suggestive of an effect, for example, the association of age and early entry into care. Also, the study might not have found the association between some of the known factors for early entry into HIV care partly because of this small sample.

Lastly, though there is a standard guide on WHO clinical staging, the assessment depends on provider level of training, experience, and available resources. Clients in this study were from a different level of clinics with an expected different level of expertise. The difference might have resulted in staging bias. However, the CD4 assay is standard across the clinics. This can be the reason for the nonconformant data for the CD4 count and WHO clinical stage in the progression of the disease.

## Recommendations

The study findings lead to several recommendations for improving early entry in care and treatment. Healthcare provider counselling should emphasize the importance of early entry in care. More time should be spent talking to newly diagnosed HIV-positive clients to provide individualized, proper counselling and education. Clients need to be thoroughly informed of their treatment options, and the consequences of each, so that they are aware of the effects of late enrolment in CTC. This study revealed that most clients do not receive one-on-one counselling; instead, they are collected as a group and given general knowledge about the medication they have been given. Implementation of this recommendation will be challenging, given that CTCs are understaffed, and the number of clients is growing. Thus, there should be deliberate efforts to increase the number of staff based on each department’s needs.

In addition to improving counselling and education, clients who feel health since they are asymptomatic should be escorted to a CTC, because asymptomatic clients do enrol late in care and treatment.

The district health management team and MOHCGEC should continue to provide HIV health education to the community, with a focus on stigma reduction and the importance of early entry in care after a positive HIV test. It appears that most clients attend a CTC as a matter of routine, because they have been told to do so, and not because they understand the advantage of continuous monitoring.

The MOHCGEC should redesign the referral form to capture more relevant information about the client to help with tracking clients who fail to attend a CTC.

We recommend that a larger quantitative study be conducted to capture a larger population of newly diagnosed PLHIV. This study should investigate clients who delayed attending a CTC and their reasons for, and perceptions about, doing so.

## Conclusion

HIV needs to be better understood in the community so that people can accept it and adhere to treatment plans, when needed. Efforts to encourage people to get back to a CTC after testing HIV-positive need to involve both sexes, and the collaboration of multisector stakeholders, and should strive to achieve maximum participation. Late entry into care and treatment programs and failure to adhere to medication after testing HIV-positive have been linked to fear and stigma. Fear and stigma both need to be addressed so that the community accepts HIV, like any other disease; clients feel free to disclose their HIV status; and PLHIV attend a CTC regularly, when necessary.

## Supporting information

S1 Data DelaytoCTC. (DTA)

S2 Data Secondarydata. (DTA)
